# Supplementary material for: New Insights on Liver-Directed Therapies in Hepatocellular Carcinoma
Source: Cancers (Basel). 2023 Dec 8;15(24):5749. doi: 10.3390/cancers15245749 (PMC10741466; doi:10.3390/cancers15245749)
Supplement: Supplementary file 1 [file cancers-15-05749-s001.zip › cancers-2726039-supplementary.pdf]

Table S1: Combination of TACE and Systemic Therapies

Clinical Trial Name	Phase	Therapy	Control	Results
**SPACE**[54]	II	DEB-TACE + Sorafenib	DEB-TACE + placebo	No difference in median OS, TTP, or macrovascular invasion/extrahepatic spread between groups.
**TACE 2**[55]	III	DEB-TACE + Sorafenib	DEB-TACE + placebo	No difference in PFS.
**TACTICS**[57]	II	TACE + Sorafenib	TACE alone	Significantly increased TACE-specific PFS in the combination group. No difference in OS.
**LAUNCH**[59]	III	TACE + Lenvatinib	Lenvatinib alone	Significantly improved OS, PFS, and objective response rate in the combination group.
**Xia et al** [58]	Retrospective	DEB-TACE + Lenvatinib	Lenvatinib alone	Significantly improved OS and PFS in combination group.
**Britten et al.** [64]	II	TACE + Bevacizumab	TACE alone	Significant increased PFS in the combination group.
**DEMAND** NCT04224636	II	Atezolizumab-Bevacizumab + TACE	Atezo-Bev prior to TACE	Ongoing
**Wang et al.** [60]	Retrospective	TACE + Lenvatinib + PD-1 inhibitor	TACE + Lenvatinib	Significantly increased OS and PFS in the PD-1 inhibitor combination group.
**Han et al.** [61]	Retrospective	TACE + TKI + ICI	TACE + TKI and TACE alone	Significantly increased median OS and PFS in the TACE + TKI + ICI group.
**EMERALD-1** NCT03778957	III	DEB or cTACE + Durvalumab followed by Durvalumab or combination of Durvalumab and Bevacizumab	TACE alone	Ongoing
**EMERALD-3** NCT05301842	III	TACE + Durvalumab + Tremelimumab with or without Lenvatinib	TACE alone	Ongoing
**IMMUTACE** NCT03572582	II	TACE + Nivolumab	Efficacy	Ongoing
**PETAL** NCT03397654	Ib	TACE + Pembrolizumab	Efficacy	Ongoing
**PI: Greten**[80] NCT03937830	II	DEB-TACE + Durvalumab + Bevacizumab + Tremelimumab	Efficacy	Recruiting
